# RETRACTED: Lee et al. Machine-Learning-Based Survival Prediction in Castration-Resistant Prostate Cancer: A Multi-Model Analysis Using a Comprehensive Clinical Dataset. *J. Pers. Med.* 2025, *15*, 432

**DOI:** 10.3390/jpm16020083

**Published:** 2026-02-02

**Authors:** Jeong Hyun Lee, Jaeyun Jeong, Young Jin Ahn, Kwang Suk Lee, Jong Soo Lee, Seung Hwan Lee, Won Sik Ham, Byung Ha Chung, Kyo Chul Koo

**Affiliations:** 1 Department of Urology, Gangnam Severance Hospital, Yonsei University College of Medicine, Seoul 06273, Republic of Korea; 2 SKTelecom, Seoul 04539, Republic of Korea; 3 Department of Urology, Severance Hospital, Yonsei University College of Medicine, Seoul 03722, Republic of Korea; 4 Department of Urology, Urological Science Institute, Yonsei University College of Medicine, Seoul 06273, Republic of Korea

The journal retracts the article “Machine-Learning-Based Survival Prediction in Castration-Resistant Prostate Cancer: A Multi-Model Analysis Using a Comprehensive Clinical Dataset” [1] cited above.

Following publication, the authors contacted the Editorial Office regarding methodological flaws in the model development and validation presented in this article [1].

In accordance with standard journal procedures, the Editorial Board evaluated the flagged issues relating to the absence of an era-stratified analysis and information leakage and confirmed that these errors compromise the overall validity of the findings. Consequently, the Editorial Board, together with the authors, decided to retract this publication [1] as per MDPI’s retraction policy (https://www.mdpi.com/ethics#_bookmark30, accessed on 13 January 2026).

This retraction was approved by the Editor-in-Chief of the *Journal of Personalized Medicine.*

The authors agree to the retraction of this article and support this action in the interest of maintaining the integrity of the scientific record. The authors apologize for any inconvenience caused to the journal and its readers.
